# Physical Medicine and Rehabilitation: From the Birth of a Specialty to Its Recognition

**DOI:** 10.7759/cureus.17664

**Published:** 2021-09-02

**Authors:** Hafid Arabi

**Affiliations:** 1 Physical Medicine, Cadi Ayyad University, Marrakesh, MAR

**Keywords:** physical medicine and rehabilitation, history, medical gymnastics, massage, physiotherapy

## Abstract

Physical Medicine and Rehabilitation (PMR) was subordinate to different specialties. Currently, it is recognized as essential to improving healthcare. How did it originate and which events impacted it? Different events have impacted the development of PMR including war, epidemic, sport, natural disasters, and the demographic and sociocultural characteristics of each country. At present, PMR is experiencing some difficulties throughout the world; we present proposals to improve its position to bring the specialty up to the specialty podium. Accidentology, sports events, and natural disasters enrich medicine in general and PRM in particular. Indeed, all these events create disability through a participatory and integrative strategy of the patients; PMR always takes up the challenge.

## Introduction and background

The involvement of Physical Medicine and Rehabilitation (PMR) in the current coronavirus disease 2019 (COVID-19) pandemic reminds us of its ever-present role in human history. Although PMR is quite old, it did not establish itself as a specialty in its own right until the middle of the 20th century. The role of war, epidemics, sport, professional, environmental, and accidental events in the development of PMR as a healthcare system cannot be overlooked. In times of peace as in times of war, the challenges of PMR are enormous. We will examine modern and ancient history to understand where PMR is today, we will discuss efforts to achieve broad contemporary acceptance, and, finally, we will discuss the research and teaching of this specialty.

## Review

We conducted a literature search using Medline, gray literature, and Scopus databases. The search terms included “Physical Medicine” or “Rehabilitation” associated with the following terms: war, infection, natural disaster, sport, origin, country, difficulty, and education. We also evaluated the references of the articles, which provided us with additional articles.

Origins of Physical Medicine and Rehabilitation

Specialties began to become more individualized in the 19th century. For example, organ pathology gave rise to neurology, cardiology, gastroenterology, etc. Physiopathology gave rise to allergology and dietetics. Pathogen gave rise to infectiology or physiology. The stages of development of the organism gave rise to pediatric and geriatrics. The study of the environment gave rise to occupational medicine, sports medicine, or tropical medicine. Investigative techniques gave rise to radiology.

The development of PMR has revolved around physical practice, rehabilitation, and instrumentation (prosthetics and orthotics). Physical practice is the set of manual techniques such as medical-gymnastics, massage, hydrotherapy, and electrotherapy and instrumental means of therapy and evaluation [1]. Vocational and functional rehabilitation uses the full range of medical, psychological, and social means to aid disabled persons (DP) [2]. For centuries, patients with disabilities were treated by different methods such as light, thermotherapy, manipulation, massage, and movement. Since 2,500 BC (ancient China), movement has been used to promote health [3-6]. Based on these data, it appears that PMR has been practiced for centuries; however, no one took the initiative to bring all these currents together until the middle of the 20th century [7]. Another new trend that has been added to the development of PMR is instrumentation. The manufacturing of assistive devices is older than previously thought. Believing that there would be life in the afterlife, Egyptians made devices for their mummies to restore bodily integrity [6]. For example, canes were discovered in the tomb of Tutankhamun confirming that the pharaoh suffered from a disorder of the lower limbs (clubfoot) [8]. Initially, this field was not that of doctors or surgeons but of craftsmen [9]. In the 16th century, artificial hands were made by blacksmiths [9]. This trend grew with the influx of wounded from the two world wars.

Both world wars resulted in many casualties. It was believed that it was more cost-effective to reintegrate the wounded through rehabilitation rather than recruiting new soldiers or even paying pensions [10]. Thus, many services were available to recover injured soldiers [7,11]. In Europe, the practice of rehabilitation medicine began to be organized after the war in 1950 [11]. The term “Rehabilitation and Functional Rehabilitation” was coined to designate this functional medicine. In the United States, the specialty “Physical Medicine and Rehabilitation” was officially created in 1947. The wars in the United States (World War II, Vietnam, Iraq, and Afghanistan) were full of experiences for PMR. C. Hertzman, a PMR doctor in the Vietnam War, was able to identify certain principles in such circumstances [12]. In Latin America, the global conflict has also favored PMR [13]. In France, functional rehabilitation became “Physical Medicine and Rehabilitation” in 1995. After the war, civil rehabilitation took over, applying the concepts developed for wounded soldiers to sick and wounded civilians and developing new approaches.

Physical Medicine and Rehabilitation and infection

It is not just war-like events; the polio epidemic [13,14], children with cerebral palsy, and patients with chronic disabling rheumatic diseases have led to the opening of rehabilitation and fitting centers to incurable patients for whom the so-called “organ specialties” no longer have anything to offer.

Currently, people are suffering from the new coronavirus. The world is fighting to get out of it safely. PMR is implicated in COVID-19 in intensive care and post-acute care. Fear of contamination has encouraged other rehabilitation procedures such as home rehabilitation with a physiotherapist, self-rehabilitation, and telerehabilitation. Medical specialties at the forefront are fighting to give patients a chance for survival. The primary mission of PMR is to give patients a chance for survival and functional recovery. “I think therefore I exist” has become “I practice therefore I exist.”

Sports Medicine and Physical Medicine and Rehabilitation

These two specialties have a rather special history. PMR has an interest in exercise and physiology [15]. At a time when war, sports, and PMR were intertwined, during the First World War, McKenzie began sports fitness programs and therapies for new soldiers and the wounded to enable them to return to combat [16]. Krusen, considered the father of PMR, was one of the first promoters of physiotherapy for injured athletes [17]. As a PMR physician and sports physician, he opened up a therapeutic avenue never before thought of for treating sports injuries as there were only physical educators and physiologists to care for the athlete. Clouter, a military doctor, joined Krusen in praising physical therapy and exercise among sportsmen [18]. Hellebrandt, a physiologist and PMR, added a room to the base built by his predecessors showing interest in exercise physiology. This action boosted sports medicine and PMR [19]. Currently, we see more and more sports injuries, all of which are treated by PMR.

Physical Medicine and Rehabilitation and natural disasters

More and more disasters are striking our globe. There is evidence that there is a lack of acute PMR care during disasters. Medical complications and disability can be avoided if PMR is involved from the beginning of the disaster [20]. The epidemiology of secondary damage during landslides and floods is poorly known [21]. Efforts have been ongoing to develop rehabilitation research and training since 2011. Thus, rehabilitation principles have been established in the event of a disaster to prevent the associated disability [22].

Physical Medicine and Rehabilitation and countries

Country-specific religious, philosophical, and socioeconomic factors have influenced the development of PMR. The health history of each country is an important building block for the development of PMR. In Morocco, intoxication with adulterated aircraft engine oil in 1959 caused paralysis simulating poliomyelitis [23]. This event led to the opening of several rehabilitation centers in Morocco thanks to the World Health Organization (WHO).

In Latin America, services began to be organized from the 1940s onwards under the direction of trauma and orthopedic surgeons following the need to treat disability following surgery or illness or injury [24]. In Europe, the Second World War played an important role [15]. Thus, PMR was established in many countries in the 1950s [24]. In 1970, care developed around disability in children (polio, cerebral palsy) and adults (amputation, stroke, pain, spinal cord injuries) [24]. Mexico has had hydrotherapy, electrotherapy, and mechanotherapy units since the 1900s [25].

The monotheistic religions maintain a positive attitude towards DP. Disability is seen as a test by which God tests the faith of the believer. DP lives in society without any discrimination, and it is a duty to help them in any way possible. Mesopotamian, Egyptian, and Greco-Roman civilizations; 19th and 20th-century industry; and, finally, globalization are part of the history from which DP draws its existence. The treatment of DP from antiquity to the present day has favored the change of concepts. The cripple and the monster in ancient civilizations has become a citizen with rights. The evolution of the concept of disability has finally given birth to the biopsychosocial model represented by the International Classification of Functioning, Disability and Health. However, technical and economic development has given rise to low and middle-income countries. This condition leads to disability and poor education and economic status. The WHO has developed a plan to address disability and recognizes PMR care as a key to coping [26].

PMR is in crisis in some continents. Africa is the second most populated continent, with more than 61% of the population living in rural areas [27]. The problem of the concentration of rehabilitation services in metropolitan areas makes poor areas inaccessible to PMR care [28]. Service managers lack training in disability [29]. In Africa, the unstable sociopolitical environment is an unfavorable factor for health, where DP are stigmatized. In such conditions, disability is often hidden from view. Currently, infectious diseases are the main source of disability [27,30].

Research and teaching

The global academic production is about 2.5 million items per year, doubling every nine years [31]. Clinical research in practice is not as simple in PMR in the age of evidence-based medicine. There are many biases, such as confusion bias due to the difficulty of comparing a technique to a placebo is one. Despite these methodological difficulties in clinical research, research is growing as a result of tools such as the movement analysis laboratory, isokinetism, urodynamics, musculoskeletal ultrasound, vestibular rehabilitation, neuropsychology, neuro-urological explorations, electro-neuro-myography, chronic pain, spasticity, orthopedic devices, and virtual rehabilitation. There are several research areas in PMR: the epidemiology of disabilities, the understanding of pathophysiological mechanisms of disabilities, the validation of metrological tools and clinical and instrumental evaluation, and, finally, the socioeconomic impacts of disabilities, therapeutic approaches, and care systems. PMR has consolidated its place in fields that have remained in the shadows for years, such as spasticity, scoliosis, neuro-urology, neuropsychology, chronic pain, and sports traumatology. The highest forms of evidence for PMR research (randomized controlled trials, systematic reviews, and meta-analyses) are increasing significantly [32]. By transforming scientific research to its advantage, PMR limits the attacks by its detractors.

Difficulties of Physical Medicine and Rehabilitation

The role of PMR physicians is to diagnose and propose evaluations of deficiencies and handicaps in various fields such as neuro-orthopedics, sports medicine, orthopedic devices, and neuro-urology, as well as to prescribe and coordinate complex rehabilitation procedures. Finally, PMR helps in the orientation and reintegration of DP and in the long-term medical follow-up. Despite the pivotal role of PMR physicians in healthcare, the specialty faces numerous challenges. PMR is a cross-cutting specialty whose diagnostic and therapeutic activities intersect with many other medical and surgical disciplines [33]. For the general public, PMR is subordinate to, or even merges with, other medical specialties such as rheumatology and neurology. The most difficult problem is semantic. The four terms (medicine-physical-rehabilitation-functional) confuse patients and often they consult a doctor whose specialty refers to their diseased organ (heart and cardiology-lung and pneumology). La Rééducation et Réadaptation fonctionnelle (RRF) is still present in some French-speaking countries, so PMR services are called RRF, a term that has been abandoned since 1995 [34]. In the United States, the term Physical Medicine and Rehabilitation is used, while in Europe, Physical and Rehabilitation Medicine is used [35]. PMR physicians in the United States do not consider “physiatry” to be the ideal term. The name of PMR physicians has been distorted by the public into a psychiatrist or a podiatrist, with the public sometimes referring to them as physiotherapists [36]. Other PMR physicians prefer to present themselves as nerve, muscle, and bone doctors. They believe that their specialty is more than physical medicine and rehabilitation and prefer rehabilitation medicine for the specialty and rehab doctors for PMR physicians [36]. Under others, they prefer rehabilitation medicine or PMR physicians. Even in writing this article, I had difficulty in referring to the PMR physician, which does not derive from the name of the specialty as it does for other specialties, and finally, we opted for “the PMR physician.” This lack of uniformity in the name does not make it easier to understand the scope of PMR.

While PMR performs innovative acts and generates resources, they are badly rated. Human resources, especially in poor countries are lacking, there are 10 PMR professionals per 1,000,000 inhabitants [37]. PMR is based on function, and the patient must be more involved in his or her own care; this approach is a source of therapeutic failure because the patient likes to be passive rather than active in his or her own care. Rehabilitation, even if well done, is prone to failure in the face of an uncooperative patient. Other problems hinder the development of this emerging specialty. Contrary to some specialties, the patient undergoing PMR has one or more interveners (PMRP, physiotherapist, psychomotor therapist, occupational therapist, etc.), who follow a slow and continuous rehabilitation process that requires time and a complex transversal medical and social organization. PMR guarantees the quality of life, not health; if the patient does not integrate this approach, he is looking for health. Finally, we note the absence of academic teaching of PMR in some countries.

Paradigm Shift

Voices are being raised to advocate for PMR involvement from the acute sector. Forward medicine is already operational in armed conflicts and natural disasters to reduce the consequences of delayed care. PMR physicians, with their medical knowledge in musculoskeletal conditions, can play the role of frontline medicine [38]. All PMR university departments throughout the world have established programs in general biomechanical-physiological as well as organ and specific knowledge of the pathologies causing disability. This training would enable PMR to deal with all eventualities in acute as well as in follow-up care and rehabilitation. Weinstein has proposed to approach both sick and healthy individuals by promoting health and fitness [39].

More Efforts: It Is Time to Act to Ensure That This Specialty Gets the Place It Deserves?

**Figure 1:**
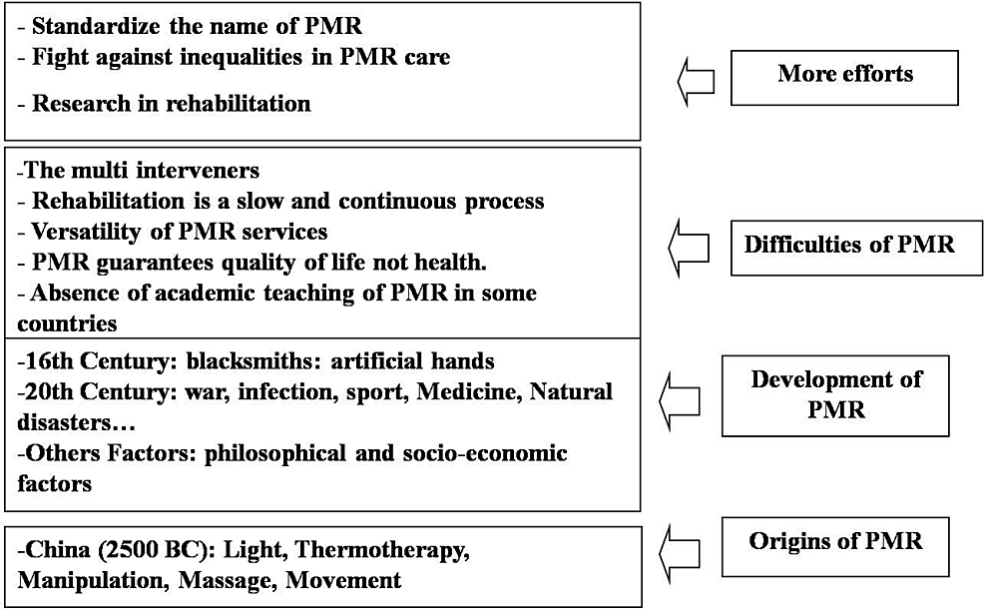
The development of the PMR since its origin and the various difficulties. PMR: Physical Medicine and Rehabilitation

The name of the specialty should be standardized for high visibility. Indeed, patients often consult PMR after seeing other specialists. Should we change the name of this specialty to conquer more ground and make our field of action better known? PMR is a specialist in function, we propose “functionologist” or “bone nerve muscle joint: BNMJ functionology” such as ORL “otorhinolarynogist.” To fight against inequalities in the care of PMR physicians, especially in mid and low-income countries, it is necessary to implement the following policies in favor of PMR physicians in these countries: teach PMR in medical schools; recruit PMR physicians in Médecins Sans Frontières, Red Crescent, and Red Cross organizations, especially in management positions; help associations of PMR physicians; facilitate the access of foreign volunteers to poor regions; benefit from assistive technologies for PMR physicians; implement scholarships for PMR physicians; and share experiences between countries. There is a need to build research capacity in rehabilitation in the military and civilian sectors and to share experiences between the two sectors. Military personnel work in a hostile environment and are exposed to more stress than civilians by participating in conflict and relief efforts during natural disasters such as floods, earthquakes, and fires. Our gaze looks to the future, but we need to examine our past to learn lessons. In closing, I paraphrase Antonis: “Let us not forget that those who do not know their history are destined to repeat it” [40]. Figure 1 summarizes the different points I made in this article.

## Conclusions

Disability is universal and spares no one. The history of humanity is full of destruction and catastrophe, yet humans have learned the best ways to preserve humankind. This flashback reveals that PMR is quite old, and wars, epidemics, sports events, and accidentology have contributed to the development of PMR as healthcare. PMR methods have played a big role in healthcare since ancient times. Despite the change in cultures and civilizations, PMR has been able to preserve itself. It has adapted while keeping its main objective: to be interested in the functional prognosis, like resuscitation, which is interested in the vital prognosis. However, it is experiencing several difficulties related to certain factors specific to each country and continent.

PMR still encounters many difficulties. More efforts should be made to give it more visibility and let it claim the place it deserves among other medical specialties. The homogenization of its name and its teaching is an obligation towards patients and other specialties. Regardless of the language or affiliation, we have a responsibility to carry the torch high to light the way for our PMR colleagues in faraway places. This torch would be a beacon to safely navigate.

.
